# A Long-Term Follow-Up Study in Immune-Mediated Thrombotic Thrombocytopenic Purpura: What Are the Outcomes?

**DOI:** 10.3390/jcm12237305

**Published:** 2023-11-25

**Authors:** Maria Addolorata Bonifacio, Daniele Roselli, Claudia Pia Schifone, Alessandra Ricco, Angelantonio Vitucci, Lara Aprile, Maria Addolorata Mariggiò, Prudenza Ranieri

**Affiliations:** 1 Department of Precision and Regenerative Medicine and Ionian Area, University of Bari Aldo Moro Medical School, 70124 Bari, Italy; m.bonifacio@studenti.uniba.it (M.A.B.); rosellidaniele94@gmail.com (D.R.); 2 Unit of Hematology and Stem Cell Transplantation, Azienda Ospedaliero-Universitaria Consorziale Policlinico, 70124 Bari, Italy; claudiaschifone24@gmail.com (C.P.S.); alessandra.ricco@policlinico.ba.it (A.R.); 61vitux@gmail.com (A.V.); 3 Hematology Unit, Presidio Ospedaliero S.G. Moscati di Taranto, 74010 Taranto, Italy; laraaprile84@gmail.com; 4 Section of Experimental and Clinical Pathology, Azienda Ospedaliero-Universitaria Consorziale Policlinico di Bari, 70124 Bari, Italy

**Keywords:** thrombotic thrombocytopenic purpura (TTP), von Willebrand factor, ADAMTS13, ADAMTS13/VWF ratio, thrombosis, biomarker, laboratory diagnostics, thromboinflammation

## Abstract

Endothelium damage triggers the multimeric protein von Willebrand factor (VWF) release and subsequent binding to platelets, which are recruited at sites of vascular injury. A complex and fragile equilibrium between circulating levels of von Willebrand factor and its metalloprotease, ADAMTS13, is responsible for the hemostatic balance. However, the presence of autoantibodies targeting ADAMTS13 results in an increase in von Willebrand factor, mainly in its ultra-large multimers. The latter lead to platelet aggregation, the formation of thrombi and microangiopathic hemolytic anemia. This pathologic condition, known as immune-mediated thrombotic thrombocytopenic purpura (iTTP), occurs with high morbidity and a high rate of relapses. In this work, the long-term follow-up of 40 patients with iTTP is reported. We assessed ADAMTS13 activity, plasmatic VWF levels and the ADAMTS13/VWF ratio, comparing iTTP relapsing patients with remitting ones. A decrease in the ADAMTS13/VWF ratio, along with a reduced ADAMTS13 activity, could serve as predictive and sensitive biomarkers of incoming relapses.

## 1. Introduction

Thrombotic thrombocytopenic purpura (TTP), also known as Moschowitz syndrome, is a rare thrombotic microangiopathy characterized by the pentad of microangiopathic hemolytic anemia, thrombocytopenia, renal dysfunction, fluctuating neurologic impairment and fever [1]. However, all five symptoms occur in less than 10% of TTP cases, while severe thrombocytopenia (<30 × 10^9^/L) and microangiopathic hemolytic anemia take place quite often during acute TTP events [2]. Sometimes, symptoms like mucocutaneous bleeding, asthenia and dyspnea may occur [3]. Therefore, TTP is a clinical emergency that requires timely diagnosis based on clinical and laboratory assessments [4]. A suspect TTP diagnosis on hemolytic patients could be driven by laboratory findings such as increased lactate dehydrogenase, reduced haptoglobin levels and presence of schistocytes in blood smear [5]. The International Society of Thrombosis and Haemostasis (ISTH) has recently highlighted the relevance of ADAMTS13 testing in order to guide therapeutic choices and TTP management [6]. Indeed, the inactivity or severe deficiency of ADAMTS13 is the pivotal laboratory parameter enabling to distinguish TTP from other thrombotic microangiopathies, especially from hemolytic–uremic syndrome (HUS) [7].

Current therapeutic strategies, including corticosteroids, rituximab and therapeutic plasma exchange (TPE), allow for the control of acute events, but are very expensive and can be administered only in specialized centers [8]. In addition, the use of caplacizumab, a humanized nanobody against VWF, is still controversial as a frontline therapy, even though it has enabled a shortened time-to-response and has reduced the risk of refractory disease [9,10].

ADAMTS13 (A disintegrin and metalloproteinase with thrombospondin type 1 motif, 13) is mainly synthetized by interstitial hepatic stellate cells, but also by platelets, endothelial cells and renal podocytes [11]. This enzyme is responsible for the cleavage of the von Willebrand factor (VWF) [12,13]. Indeed, a strong reduction in ADAMTS13 enzymatic activity, with subsequent dysregulation of the VWF/ADAMTS13 axis, is the basis of TTP onset [14]. The same axis imbalance occurs during chronic thromboembolic pulmonary hypertension, as recently reported by Newnham and colleagues [15]. ADAMTS13 deficiency could be caused by acquired autoantibody-mediated functional inhibition (iTTP) [16] or by more than 200 inherited mutations within the ADAMTS13 locus, causing a congenital disease (cTTP) also known as Upshaw–Schulman syndrome [17,18]. A cTTP diagnosis must be confirmed by genetic analyses to identify ADAMTS13 mutations in homozygous or heterozygous states [19]. Conversely, the detection of autoantibodies against ADAMTS13 through an ELISA or a Bethesda assay provides evidence of iTTP [20]. The latter accounts for the 95% of all TTP diagnoses. Nevertheless, in both cases, the reduced proteolysis of VWF high molecular weight multimers triggers severe thrombotic events on activated or injured endothelium [21]. In addition, high plasmatic levels of VWF contribute to hemostatic imbalance and ultimately to thrombosis [22,23,24].

However, an increase in VWF only indicates an inflammatory acute phase response, thus it cannot be exploited as a specific biomarker of TTP [25]. Indeed, the increased production of high molecular weight VWF multimers is affected by blood group, pregnancy, aging and physical and emotional stress [26,27,28,29]. Furthermore, VWF levels may increase during infections, cancer and hypoxia [30,31,32]. However, an increased VWF is not enough to lead to TPP. Hence, iTTP could be triggered by several conditions, such as an abnormal immune response to viral infections, an autoimmune disorder, the onset of a malignant disease, a bone marrow transplant or the assumption of chemotherapeutic drugs [33]. In most iTTP cases, it is hard to find the triggering factor because of the wide spectrum of clinical presentations accompanying the acute events. Indeed, iTTP patients may experience fever, blurred vision, gastrointestinal symptoms, headache, neurological disorders, seizures, myalgia and bruises or purpura [34]. In this respect, severe bleeds due to thrombocytopenia are uncommon, although petechiae, epistaxis or mild-to-moderate cutaneous bleedings may occur [35]. Considering the high variability of symptoms and the severeness of this clinical condition, predictive models were developed (e.g., Bentley score, French score and PLASMIC score) to support emergency units to distinguish TTP from other thrombotic microangiopathies, as well as to quickly decide whether or not to perform TPE. These scoring systems were based on clinical signs (i.e., thrombocytopenia and renal dysfunction) and readily available laboratory tests [36]. Among them, the PLASMIC score is the most widely used model to predict a severe ADAMTS13 deficiency, developed by Bendapudi and coworkers [37]. Together with the careful assessment of clinical signs, the PLASMIC score is a powerful tool when testing ADAMTS13 [38]. It is based on seven parameters, i.e., platelet count (<30 × 10^9^/L), creatinine level (<2 mg/dL), hemolysis signs (reticulocyte count > 2.5%, undetectable haptoglobin, indirect bilirubin > 2mg/dL), MCV (<90 fL), INR (<1.5) and associated conditions (i.e., absence of active cancer and no transplants received). Each parameter accounts for 1 point, enabling the clinician to stratify patients in high risk (6–7 score), intermediate risk (5 score) and low risk (0–4 score) of ADAMTS13 depletion. An intermediate risk score warrants a closer observation of the patient, while the high risk scores make patients suspected of TTP, enabling a start to their treatment [39]. Nevertheless, the detection of a severe ADAMTS13 activity, of less than 10%, is the laboratory finding that confirms current clinical diagnoses of TTP [40]. Indeed, iTTP arises from autoantibodies binding ADAMTS13, hindering its enzymatic activity or enhancing its clearance [41,42]. Nevertheless, in 15–22% of iTTP patients, ADAMTS13 functionality remains low, even though a platelet count normalization and a remission state is confirmed [43]. Given the high risk of fatal complications associated to iTTP, the close monitoring of these patients is required. Hence, a systematic assessment of ADAMTS13-related parameters is strongly needed in order to optimize relapse management [44].

In this respect, the evaluation of the ADAMTS13/VWF ratio has been reported for chronic thromboembolic pulmonary hypertension and acute kidney injuries in COVID-19 patients [45,46] and for portal vein thrombosis in cirrhotic patients [47].

In this work, we report the follow-up of 40 patients affected by iTTP, from their first acute event to remission or relapse. Diagnostic laboratory parameters VWF, ADAMTS13 activity, ADAMTS13/VWF ratio and ADAMTS13 inhibitor have been assessed over time, studying their potential to be predictive biomarkers of iTTP relapse.

## 2. Materials and Methods

### 2.1. Patients Follow-Up

From 2014 to 2023, we followed-up with 40 patients with a first diagnosis of iTTP, who were admitted to the Hematology Units of the Hospitals in Bari and Taranto during routine clinical practice.

The diagnosis of iTTP was performed according to the current ISTH guidelines [6], calculating the PLASMIC score and assessing laboratory parameters (i.e., VWF, ADAMTS13 activity, ADAMTS13/VWF ratio and ADAMTS13 inhibitor). A 0 to 4 PLASMIC score has been considered as a low risk, while a 5 score has been considered as an intermediate risk and a 6 to 7 score has been considered as a high risk of ADAMTS13 severe deficiency. Since the PLASMIC score was introduced as a clinical prediction tool only in 2017, 11 patients were evaluated according to previous guidelines [48].

All patients with a high pre-test probability of TTP diagnosis, who were then confirmed with severe deficiency in ADAMTS13 activity, started TPE and/or corticosteroids, continuing until recovery of platelet count.

After the acute event, all patients were scheduled for monthly follow-ups for the first three months, then for routine check-ups every three months for the first year, then for check-ups every six or twelve months. A temporal variation of ±1 month was allowed for each time-point in the closest monitoring phases and ±2 months for the three-month, six-month or twelve-month monitoring phases.

### 2.2. Laboratory Methods

Blood samples were collected in citrated plasma tubes (BD Vacutainer^®^, Milan, Italy) before performing TPE. Samples were immediately processed and used within 24 h for laboratory panel testing to detect iTTP (VWF, ADAMTS13 activity and ADAMTS13 inhibitor).

ADAMTS13 activity was assessed using a chromogenic enzyme-like immunosorbent assay (ELISA), using GST-VWF-73 as substrate (Technozym^®^ ADAMTS13 Activity, Technoclone Herstellung von Diagnostika und Arzneimitteln GmbH, Vienna, Austria), reading optical density at 450nm, as recommended by the manufacturer. Results were expressed as percentages (normal values range 0.4–1.3 IU/mL, limit of quantification 0.0071 IU/mL).

ADAMTS13 functional inhibitor activity was measured using a mixing test exploiting patient plasma as well as normal plasma in 1:1 ratio and incubating for 2 h at 37 °C. The residual enzyme activity is measured after incubation. Inhibition of 50% of normal plasma ADAMTS13 activity by undiluted patient plasma is defined as one Bethesda unit (BU). The presence of an ADAMTS13 inhibitor is reported when ≥0.5 BU/mL is detected.

The VWF antigen was measured with the latex immuno-turbidimetric method (von Willebrand Factor Antigen/HemosIL TM, Instrumentation Laboratory, Milan, Italy). Results were expressed as percentages (normal values range 60–150%, limit of quantification 2.2%).

### 2.3. Statistical Analyses

Statistical analyses were performed using R software, version 4.0.1. (R Development Core Team) Univariate analysis between the levels of ADAMTS13, ADAMTS13/VWF ratio and VWF of two different iTTP patient groups (relapsing vs. remitting) was performed through Spearman’s correlation test. A *p* value of less than 0.05 was considered statistically significant.

ROC curves were built to find the optimal cut-off to distinguish relapsing and remitting patients. Then, the cut-off was exploited to perform a Cox regression analysis and to calculate the hazard ratio.

## 3. Results

**Figure 1:**
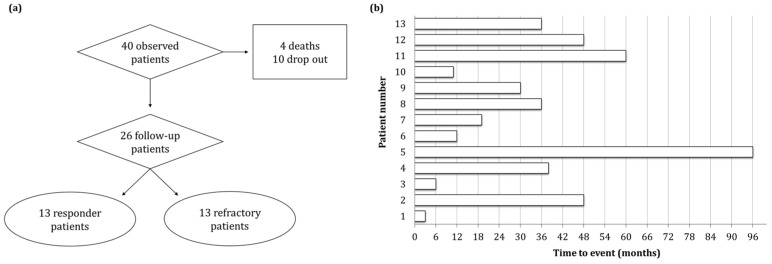
Flow chart description of the patients monitored during the follow-up period (**a**). Relapse-free time for each of the 13 patients followed-up with in this study, identified by a number between 1 and 13 (**b**).

Figure 1a depicts the patients monitored in this follow-up study. More than half the patients had other comorbidities. Thus, over ten years, 4 patients passed away (10%) and 10 (25%) dropped out of the follow-up, mainly because of the emergence of the COVID-19 pandemic. Within the remaining 26 patients still in follow-up, 13 patients responded to therapy and were in durable remission, while the remaining 13 were refractory and experienced a TTP relapse over a median time of 48 months (Figure 1b). One year before iTTP onset, four patients experienced a relapse. More than 2 years later, six patients experienced a second additional relapse.

Table 1 reports the characteristics of 26 patients with acute iTTP, followed-up with during this ten-year study. The patients’ median age was 49 years (range 29–59 years), while 9 were men (23%) and 31 women (77%). All of them were characterized by microangiopathic hemolytic anemia, severe thrombocytopenia (<30 × 10^9^/L) and ADAMTS13 activity of less than 5%. The median level of VWF was 210 ± 45%, while ADAMTS13 inhibitor ranged from 1 to 105 BU/mL. In 55% of patients, we found low ADAMTS13 inhibitor titers (ranging from 1 to 5 BU/mL), while in 45% we observed high titers (ranging from 6 to 105 BU/mL). Four patients (15%) had the predisposing HLA-DRB1*11 polymorphism.

Initially, all patients were treated with conventional therapy, i.e., TPE and immunosuppressive drugs (steroids and/or rituximab) until platelet count recovery. Patients with severe symptoms, or those refractory to conventional therapy, were treated with caplacizumab [49]. Clinical remission was declared when a persistent recovery of platelet count (>150 × 10^9^/L^−^^1^) was reported for 30 days after therapy interruption, as reported by Sukumar et al. [50]. Relapse was declared when, during a clinical remission, initial symptoms such as anemia or thrombocytopenia occurred, as well as in presence of abrupt or slowly progressive worsening of clinical status, or insidious alterations in laboratory values (i.e., ADAMTS13 activity < 10%) [51].

**Figure 2:**
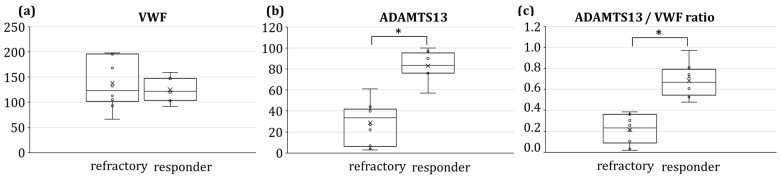
Boxplots relevant to plasmatic levels of VWF (**a**), ADAMTS13 activity (**b**) and ADAMTS13/VWF ratio (**c**) in refractory and responder patient groups. * *p* < 0.05.

All relapses occurred with plasmatic ADAMTS13 inhibitor titers ranging from 1 to 13 BU/mL except for one patient, who showed an ADAMTS13 inhibitor titer of 116 BU/mL at the second relapse event. No statistically significant difference was found between ADAMTS13 inhibitor titers belonging to responders and to refractory patients (*p* > 0.05). Similarly, Figure 2a shows that VWF percentages did not differ in the two patient groups, being 123% (IQR 81) vs. 121.5% (IQR 42) in refractory and responder groups, respectively. On the other hand, the values of ADAMTS13 activity and the ADAMTS13/VWF ratio (Figure 2b,c) were statistically different between refractory and responder patients (*p* = 4.6 × 10^−6^ and 1.3 × 10^−5^, respectively). Indeed, ADAMTS13 activity was equal to 33.5% (IQR 30) in the refractory group, while it was 83.5% (IQR 16.5) in the responder group. As far as the ADAMTS13/VWF ratio is concerned, the refractory group had a 0.23 (IQR 0.24) median value, which was one-third of that relevant to the responder group, i.e., 0.67 (IQR 0.17).

Furthermore, the calculation of Spearman’s coefficients confirmed the correlation between refractory/responder states and ADAMTS13 activity values, as well as those of the ADAMTS13/VWF ratio (Table 2). No correlation was found for VWF and ADAMTS13 inhibitor.

**Figure 3:**
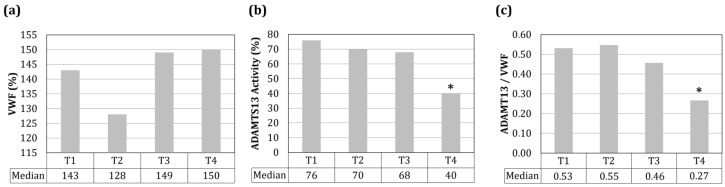
Outcomes of the laboratory analyses performed at four subsequent timepoints (T1–T4) before relapse events in the 13 refractory patients group. WVF (**a**), ADAMTS13 activity (**b**) and ADAMTS13/VWF ratio (**c**). * *p* < 0.05.

Analyzing subsequent timepoints (T1–T4) in the refractory patients group, median values of VWF, ADAMTS13 and ADAMTS13/VWF ratio could be compared (Figure 3). The timepoints T1–T4 varied according to each patient’s individual history, but followed the experimental design reported in Section 2.1. It is worth noting that, immediately before relapses, the values of VWF, ADAMTS13 activity and the ADAMTS13/VWF ratio were 150%, 40% and 0.27, respectively (Figure 3, T4). Furthermore, the values of ADAMTS13 activity and the ADAMTS13/VWF ratio were significantly different at T4 as compared to T1, T2 and T3 (* *p* < 0.05).

Moreover, the values of ADAMTS13 activity and the ADAMTS13/VWF ratio, collected for responder and refractory patients, have been further studied in an attempt to identify potential cut-off values to distinguish the two patient groups. Although the patients number was low (*n* = 26), an ROC curve was built for ADAMTS13 activity and ADAMTS13 activity/VWF ratio. The optimal cut-off value for ADAMTS13 activity resulted in a value equal to 57% (AUC = 0.99), resulting in 100% sensitivity, 90% specificity and 94.4% accuracy. Concerning the ADAMTS13/VWF ratio, a cut-off of 0.48 (AUC = 1) was associated with 100% sensitivity, 100% specificity and 100% accuracy. Furthermore, considering the obtained 0.48 cut-off, the ADAMST13/VWF ratio was categorized to refractory/responder state and a Cox regression analysis was attempted, showing that the hazard ratio for relapse occurrence is 8.65 times higher in patients with an ADAMST13/VWF ratio < 0.48 (*p* = 4.0 × 10^−6^).

## 4. Discussion

This ten-year follow-up study on iTTP patients induced us to rethink this disease, considering it as a chronic condition [52]. The monthly monitoring of ADAMTS13 activity within the first three months after TTP onset allowed us to identify those patients with slow recovery rates, whose ADAMTS13 activity values stayed lower than 20%. These refractory patients were low-responders to conventional therapy, due to high titers of ADAMTS13 inhibitors. Thus, they were selected for rituximab treatment, achieving a stable platelet count and an effective suppression of autoimmune response. The collected data showed that circulating ADAMTS13 inhibitor levels ranged from 1 to 105BU/mL. Furthermore, levels higher than 5 units were not always associated with relapse (*p* = 0.68). These observations highlight the need to further investigate immune-mediated pathogenic mechanisms triggering TTP.

A strong association between HLA loci, the occurrence of autoantibodies and the onset of autoimmune diseases was already described by Amin and coworkers [53]. In this respect, the alleles HLA-DRB111 and/or HLA-DQB103 have been associated with a higher risk of TTP development, while the allele HLA-DRB104 appears to be protective. These alleles encode surface proteins that present ADAMTS13 to CD4^+^ T lymphocytes, thus driving tolerogenic mechanisms involved in iTTP [54]. In addition, infectious agents may trigger iTTP through activation of cross-reactive CD4^+^ T lymphocytes [55].

Beyond genetic and environmental triggers, aging may contribute in reducing ADAMST13 functionality, i.e., through the lack of Zn^2+^ ions, which are required for enzymatic activity, as well as for an effective antiviral immunity [56,57,58,59].

On the other hand, VWF plays a pivotal role in thromboinflammation, a complex network of processes involving endothelial damage, inflammatory cascade activation and cytokine release [60]. Immune cells and platelet activation drive thromboinflammation, causing microvascular thrombosis, hence a wide number of diseases [61]. VWF is responsible for the recruitment of platelets on injured endothelial surfaces, providing a plethora of adhesive binding sites. VWF is released from Weibel–Palade bodies (WPBs) by endothelial cells and platelet α-granules, in which it is stored in the form of high molecular weight multimers [32]. WPB exocytosis starts primary hemostasis and induces vasoconstriction [62,63,64]. A reduced ADAMST13 activity might not be able to cleave enough high molecular weight VWF multimers, paving the way to thrombosis. Therefore, the effectiveness of TPE and immunosuppressive therapies is based on the removal of VWF high molecular weight multimers, as well as of the autoantibodies targeting ADAMTS13 [65]. In addition, ADAMTS13 supplementation results in a good platelet recovery. Indeed, all patients in the acute phase were effectively treated with TPE and/or corticosteroids. Similarly, the treatment with rituximab led to platelet stabilization and to a dramatic drop in the ADAMTS13 inhibitor. Furthermore, patients treated with caplacizumab also achieved clinical remission.

These data, as well as the adopted strategies, emphasize the opportunity to target dysfunctional immune cascades to restore effective ADAMTS13 activity and reach clinical stabilization of the patients. The complex equilibrium between VWF and ADAMTS13 makes it compulsory to study both of them, since ADAMTS13 deficiency alone, or its bare functional inhibition, is not enough to explain TTP relapse and remission states [43]. Indeed, decreased ADAMTS13 activity (<5%) was substantially asymptomatic. Moreover, the patients herein studied showed physiological VWF levels (~143%), but they were continuously increasing (up to 198%) before relapse, causing a higher susceptibility to infections, autoimmunity or malignancy. No severe thrombocytopenia or significant clinical symptoms (i.e., stroke, comatose status, transient neurological alterations, seizures) were reported during pre-relapse phases.

Conversely, when the ADAMTS13/VWF ratio was <0.01 and a lower ADAMTS13 level matched with the reappearance of anti-ADAMTS13 antibodies, a reassessment of the patient’s clinical condition was required. In cases of relapses confirmed by severe ADAMTS13 deficiency, the exploitation of immunosuppressors (e.g., rituximab) in combination with caplacizumab resulted effective. Right before relapse (T4), our patients had a reduced ADAMTS13/VWF ratio (Figure 3c) as compared with the values obtained before. These data indicate the persistence of dysfunctional mechanisms, even without causing symptoms. Thus, since ADAMTS13 deficiency alone or the inhibitor titers are not enough to explain and predict acute events [66], the ADAMTS13/VWF ratio seems a more appropriate disease marker. Given the mild nature of relapses, as compared to the onset of TTP events, the need for sensitive, predictive biomarkers should drive a deeper exploration of ADAMTS13-related parameters, in particular right before acute relapses. Indeed, at this time, our patients showed platelet count and hematocrit parameters within the physiological ranges, thus clinicians had no suspicion of the incoming relapse. However, in our diagnostic routine, we assessed the ADAMST13/VWF ratio, considering it as a biomarker of chronic dysregulated endothelium, which is in agreement with literature data [47]. In this respect, scheduling check-ups at shorter intervals could be crucial for timely therapeutic intervention.

Our findings suggest that ADAMST13/VWF ratio is as effective as ADAMST13 activity in predicting TTP relapses, although a validation on a larger number of patients is required. Preventive immunosuppression may be used to achieve ADAMTS13 recovery, reducing the risk of clinical relapses in patients with an ADAMST13/VWF ratio < 0.48 [67,68].

## 5. Conclusions

In this study, a real-life follow-up of 40 patients with iTTP, spanning from 2014 to 2023, is described. The reported data emphasize the importance of promptly and reliably detecting refractory patients. Although VWF titers did not statistically differ between the two patients groups, their assessment indicates a silent proinflammatory state and a condition of endothelial activation that should be closely monitored by the clinician. Combining the information about VWF and its protease in the ADAMTS13/VWF ratio, an imbalance that persists beyond acute disease stages can be detected, providing valuable insights into this delicate hemostatic axis.

Collecting data on a higher number of patients over time, ADAMTS13 activity and the ADAMTS13/VWF ratio could become valuable biomarkers for iTTP risk stratification, early detection of relapses and prediction of therapy outcomes.

## Figures and Tables

**Table 1:** Characteristics of the tested patients (*n* = 26).

Parameter	Characteristics	Percentage (%)
Median age (IQR)	49 (29–59)	
Gender (M:F)	9:31	23:77
PLASMIC Score ^1^	7 (*n* = 10)	38
	6 (*n* = 5)	19
	n.a. (*n* = 11)	42
Thrombosis	9/26	35
Bleeding	17/26	65
Therapy	Steroids (*n* = 10)	38
	TPE (*n* = 20)	77
	Caplacizumab (*n* = 5)	19
	Vincristine (*n* = 3)	11
	Rituximab (refractory) ^2^ (*n* = 5)	19
	Rituximab (effective) (*n* = 12)	46
Comorbidities	Infection (EBV, *E. pylori, S. aureus, K. pneumoniae*) (*n* = 4)	15
	Cancer (prostate, breast, medulloblastoma) (*n* = 3)	11
	Diabetes mellitus type II (*n* = 1)	4
	Angina pectoris (*n* = 1)	4
	Autoimmune disorders (*n* = 5)	23
Other parameters	Fever (*n* = 3)	11
	Pregnancy (*n* = 1)	4
	Neurological symptoms (*n* = 13)	50
	Renal impairment (*n* = 3)	11
	HLA-DRB1*11 (*n* = 4)	15

^1^ PLASMIC score: 0–4 low, 5 = intermediate, 6–7 high risk of severe ADAMTS13 deficiency. ^2^ Rituximab refractory consists of the absence of an increase in platelet count (<50 × 10^9^/L^−1^) associated with increased LDH, after five sessions of TPE. EBV: Epstein–Barr Virus. n.a.: not assigned.

**Table 2:** Univariate analysis performed through Spearman’s correlation test.

Laboratory Parameter	Spearman’s r Coefficient	*p*-Value
ADAMTS13	0.84	1.3 × 10^−5^
ADAMTS13/VWF ratio	0.86	4.6 × 10^−6^
VWF	−0.13	0.61
ADAMTS13 inhibitor	−0.11	0.68

## Data Availability

All reported data are herein available. Raw data are available from the corresponding authors, on reasonable request.
